# Towards Red Emissive Systems Based on Carbon Dots

**DOI:** 10.3390/nano11082089

**Published:** 2021-08-17

**Authors:** Spyridon Gavalas, Antonios Kelarakis

**Affiliations:** UCLan Research Centre for Smart Materials, School of Natural Sciences, University of Central Lancashire, Preston PR1 2HE, UK; sgavalas@uclan.ac.uk

**Keywords:** fluorescence, carbon dots, red-emitting, bioimaging, light-emitting diodes

## Abstract

Carbon dots (C-dots) represent an emerging class of nontoxic nanoemitters that show excitation wavelength-dependent photoluminescence (PL) with high quantum yield (QY) and minimal photobleaching. The vast majority of studies focus on C-dots that exhibit the strongest PL emissions in the blue/green region of the spectrum, while longer wavelength emissions are ideal for applications such as bioimaging, photothermal and photodynamic therapy and light-emitting diodes. Effective strategies to modulate the PL emission of C-dot-based systems towards the red end of the spectrum rely on extensive conjugation of sp^2^ domains, heteroatom doping, solvatochromism, surface functionalization and passivation. Those approaches are systematically presented in this review, while emphasis is given on important applications of red-emissive suspensions, nanopowders and polymer nanocomposites.

## 1. Introduction

The dynamic presence of carbon dots (C-dots) in the field of nanoemitters over the last 15 years is directly related to their unique combination of three desired characteristics: inexpensive preparation, nontoxic nature and superior photophysical properties in terms of light absorption, chemiluminescence, electroluminescence, phosphorescence and up-conversion [1,2,3,4,5]. Typically, C-dots are spherical nanoparticles with size 2–20 nm that are composed of C, O and H along with heteroatoms such as N, P and S.

Their carbogenic cores can be predominantly amorphous or highly graphitic in nature [6], while the term graphene quantum dots (GQDs) is typically reserved for nanodisks of single- or few-layered graphene sheets [7]. In that sense, GQDS are considered as a distinct subgroup of C-dots and are synthesized by various strategies including chemical vapor deposition on hexagonal boron nitride substrates [8], all-organic synthesis starting from polyphenylene dendrimers [9] and ring opening of fullerenes adsorbed to Ru metal terraces [10]. Alternatively, GQDs can be prepared by oxidation or electrooxidation of carbon-rich sources such as coal [11], carbon black [12], graphite [13], carbon fibres [14] and carbon nanotubes (CNT) [15].

Well-defined C-dots are prepared at a large scale following hydrothermal, microwave and calcination protocols performed on a wide range of feedstock materials, including renewable and abundant resources such as crude biomass [16], coffee beans [17], fruit juice [18], etc. Pyrolytic decomposition of suitable precursors gives rise to molecular fluorophores that are embedded within the carbogenic cores or are adsorbed on their surfaces [19,20]. For example, thermal treatment of urea and citric acid (CA) generates blue-emissive citrazinic acid and green-emissive 4-hydroxy-1H-pyrrolo(3,4-c) pyridine-1,3,6(2H,5H)-trione [21].

The PL properties of aqueous solutions of C-dots have been systematically investigated with emphasis on theragnostics [22], gene delivery [22], drug delivery [23], antimicrobial applications [24], bioimaging [25] and biosensing [26]. At the same time the PL properties of polymer-based nanocomposites [27,28] and powder formulations based on C-dots [29,30] are directly relevant to nanoforensics [29,30], light-emitting diodes [31] and catalytic platforms [32].

In contrast to conventional semiconducting quantum dots that are based on heavy metals, C-dots are considered as nontoxic, biocompatible and environmentally benign nanomaterials. Nevertheless, it has been demonstrated that surface functionalization can dramatically modify their cytotoxicity as well as cell uptake and intercellular trafficking [33,34].

Although a large variety of blue/green-emissive C-dots with advanced PL properties have been developed, it has been demonstrated that extensive π-conjugated domains, high levels of surface oxidation, incorporation of heteroatoms such as N, S and P and solvatochromism can give rise to red-shifted emissions [35,36]. It has been recently suggested that the red/NIR-emissive signals recorded on C-dot based systems stem from organic-like dye molecules encapsulated within carbonaceous nanostructures and might not be related to the intrinsic properties of nanoparticles [37]. This approach, however, might not explain the behaviour observed in GQDS and in related systems not prepared via pyrolysis. There is general consensus that pyrolytic treatments of precursor molecules give rise to complex mixtures of carbonaceous nanoparticles, crosslinked systems and organic molecules, necessitating further systematic studies in order to accurate deconvolute and identify the emissive contributions of each one of those components.

In this review we present promising strategies for the synthesis of C-dot-based systems with predominantly red-shifted optical signals. Particular attention is given to the preparation of red-emissive liquid suspensions, polymer nanocomposites and nanopowder formulations. The distinct advantages of those nanomaterials in emerging applications such as bioimaging, biosensing, photothermal and photodynamic therapy, light-emitting diodes, pollutant detection and nanoforensics are highlighted.

## 2. Discussion

### 2.1. Strategies to Generate Red-Shifted C-Dots

The main strategies that give rise to red-shifted C-dots rely on: (i) heteroatom doping, (ii) extensive conjugation length, (iii) surface functionalization and passivation and (iv) solvatochromism.

#### 2.1.1. Heteroatom Doping

Heteroatom doping is a well-explored strategy to modify the electronic and optical properties of C-dots, ultimately leading to red-shifted C-dots [38]. It has been supported that N doping can reduce the bandgap and improve bandgap uniformity of C-dots, thus enhancing red emissions with a narrow full width at half maximum. Moreover, B and S doping leads to reduced nonradiative recombinations, in essence intensifying red signals. Incorporation of La results in local structural rearrangements, increased charge transfer and ultimately stronger red emissions [35].

**Figure 1:**
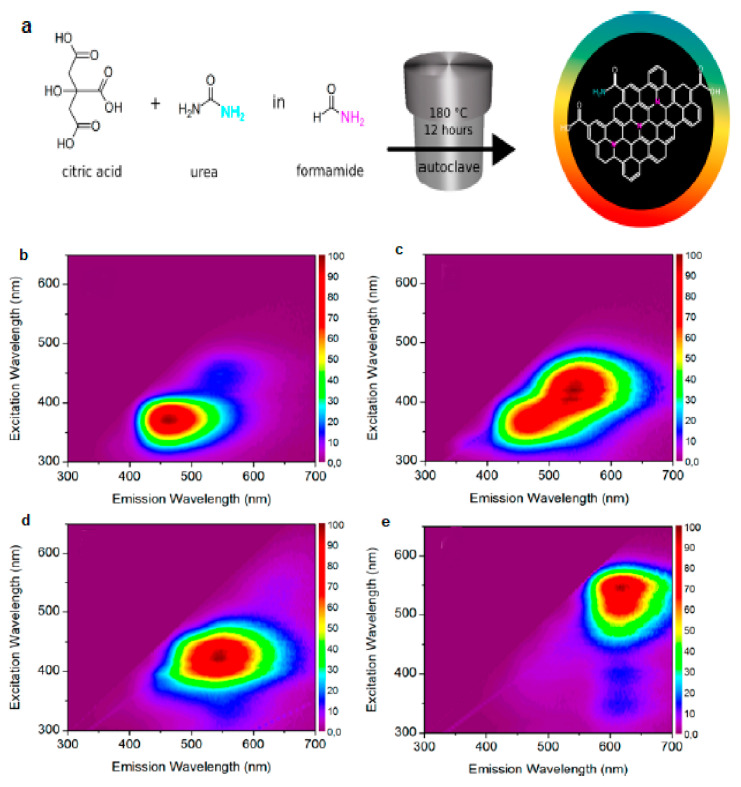
(**a**) Schematic illustration of the synthesis followed to generate a family of C-dots from CA/urea in formamide. (**b**–**e**) Fluorescence excitation−emission map of: (**b**) b-C-dots, (**c**) g-C-dots, (**d**) y-C-dots and (**e**) r-C-dots. Reprinted (adapted) with permission from [39]. Copyright 2021 American Chemical Society.

**Figure 2:**
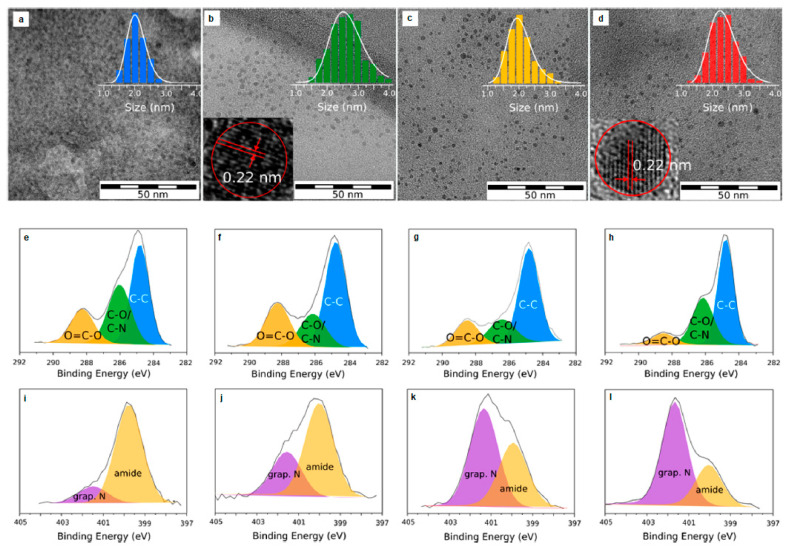
TEM images of: (**a**) b-C-dots, (**b**) g-C-dots, (**c**) y-C-dots and (**d**) r-C-dots with diameter histogram and HR-TEM images for g-C-dots and r-C-dots in the inset. High-resolution C 1s and N 1s XPS spectra for (**e**,**i**) b-C-dots; (**f**,**j**) g-C-dots; (**g**,**k**) y-C-dots and (**h**,**l**) r-C-dots. Reprinted (adapted) with permission from [39]. Copyright 2021 American Chemical Society.

To that end, Hola et al. [39] synthesized N-doped graphitic C-dots following the solvothermal treatment of urea and CA in formamide (Figure 1a) that were subsequently subjected to column chromatography yielding blue- (b-C-dots), green- (g-C-dots), yellow- (y-C-dots) and red- (r-C-dots) emissive fractions as shown in Figure 1b,c,d,e, respectively. In water, at λ_e__x_ = 440 nm, the QY of b-C-dots, g-C-dots, y-C-dots and r-C-dots were 13.3%, 10.0%, 11.6% and 4.0%, respectively. Transmission electron microscopy (TEM) imaging suggested that the average diameter (d_av_) of these C-dots was 2–3 nm (Figure 2a–d). X-ray photoelectron spectroscopy (XPS) indicated a lower concentration of oxygen, hydroxyl and carboxyl groups and an increased content of graphitic nitrogen for y-C-dots and r-C-dots (Figure 2e–l), while nitrogen stemming from surface amide groups was dominant in b-C-dots and g-C-dots. Fourier transform infrared spectroscopy (FT-IR) confirmed the presence of carboxylic C=O bonds and surface C−N bonds for b-C-dots and g-C-dots, whereas the most pronounced FT-IR peaks were ascribed to C−N= and C=N bonds for y-C-dots and r-C-dots, verifying the dominant role of graphitic nitrogen for the red-shifted fractions.

Moreover, Ding et al. [40] fabricated red graphitic C-dots (QY = 28% at λ_e__x_ = 533 nm) with d_av_ = 4.6 nm by means of solvothermal treatment of lemon juice in ethanol (λ_ex_ stands for excitation wavelength). The PL spectra of these C-dots suggested λ_ex_-dependent behaviour, with the maximum of their emission peak at λ_ma__x_ = 615 nm. FT-IR suggested the presence of O–H, N–H, –COOH and C–O on their surface, while C=C/C=N and C–N vibrations were attributed to the polyaromatic structures of the carbogenic cores. XPS confirmed the presence of C–C/C=C, C–N, C–O, C=O/C=N and COOH groups along with the presence of pyridinic C–N–C, pyrrolic C_2_–N–H and graphitic N–C_3_ groups.

Capitalizing on similar concepts, Guo et al. [41] used various ratios of two types of functionalized polythiophene derivatives to prepare a series of graphitic C-dots with tuneable PL emissions ranging from blue to near infrared (NIR). All C-dots consisted of C, O and S, while the N content was higher for the red-shifted fractions. Pan et al. [42] synthesized graphitic C-dots with d_av_ = 6.8 nm, through microwave heating of CA and formamide solution in an autoclave with QY close to 11.9%, 16.7% and 26.2% at λ_ex_ = 360 nm, 450 nm and 540 nm, respectively. Likewise, Pan et al. [43] followed microwave-assisted heating of glutathione formamide to synthesize graphitic NIR-emitting C-dots with d_av_ =2.9 nm and QY = 16.8% at λ_e__x_ = 420 nm.

**Figure 3:**
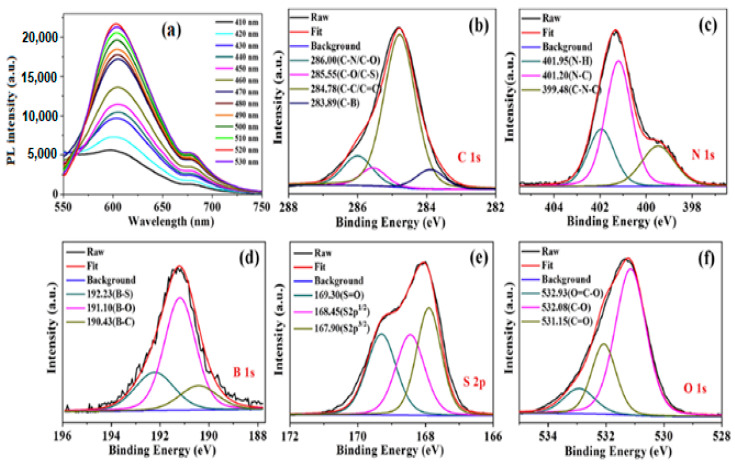
(**a**) PL spectra of NBS-C-dots with λ_ex_ ranging from 410 nm to 530 nm. C 1s (**b**), N 1s (**c**), B 1s (**d**), S 2p (**e**), and O 1s XPS spectrum (**f**) of NBS-C-dots. Reprinted (adapted) with permission from [44]. Copyright 2021 Elsevier.

In terms of multiheteroatom doping, Huang et al. [44] followed a hydrothermal method using 3-aminobenzeneboronic acid and 2,5-diaminobenzenesulfonic acid as precursors to synthesize N, B and S codoped C-dots (NBS-C-dots) with d_av_ = 2.8 nm and QY = 11.6% at λ_e__x_ = 520 nm. NBS-C-dots showed λ_ex_-independent behaviour with λ_max_ = 605 nm for 410 nm ≤ λ_ex_ ≤ 530 nm (Figure 3a). The high-resolution C 1s XPS spectrum indicates the presence of C–B (283.89 eV), C–C/C=C (284.78 eV), C–O/C–S (285.55 eV) and C–N/C–O (286.00 eV) (Figure 3b). The N 1s XPS spectrum can be deconvoluted into three main peaks at 401.95 eV, 401.20 eV and 399.48 eV, which are related to N–H, C–N and C–N–C, respectively (Figure 3c). The B 1s XPS spectrum reveals three peaks at 192.23 eV, 191.10 eV and 190.43 eV, corresponding to B–S, B–O and B–C, respectively (Figure 3d). The S 2p XPS spectrum indicates two peaks at 167.90 eV and 168.45 eV, which relate to the S 2p3/2 and S 2p1/2 spectra of the C–S–C bond, and one peak at 167.9 eV, which is attributed to –C–SO_x_– (x = 2, 3, and 4) (Figure 3e). The O 1s XPS spectrum shows three peaks at 532.93 eV, 532.08 eV and 531.15 eV, ascribed to O=C–O, C–O and C=O, respectively (Figure 3f). FT-IR spectroscopy suggests the presence of O–H, N–H bonds, -SCN- groups and C=O, C=C, C–N, C–C, C–S and C–O–C bonds, along with B–O, B–O–H, C–B and B–O–H bonds.

Xu et al. [45] followed a hydrothermal treatment of L-methionine and urea using water and dimethylformamide (DMF) as the solvent to produce red- (N,S-doped) and blue-emitting (N-doped) graphitic C-dots, respectively. Miao et al. [46] followed a hydrothermal treatment of CA and thiourea in acetone to synthesize graphitic red-emitting N,S-doped C-dots with QY = 22% in water. Ge et al. [47] followed a hydrothermal treatment of polythiophene phenylpropionic acid (PPA) to synthesize graphitic, red-emissive S-doped C-dots with d_av_ = 10 nm exhibiting λ_ex_-independent properties with QY = 2.3%. Yang et al. [48] followed a hydrothermal treatment of 2,5-diaminobenzenesulfonic acid to synthesize graphitic, red-emissive N,S-doped C-dots with d_av_ = 4.9 nm and QY = 2.7% at λ_ex_= 500 nm.

In situ chromophore doping can lead to red-shifted C-dots by introducing new electron transition pathways that alter their highest occupied molecular orbital and lowest unoccupied molecular orbital (HOMO–LUMO) levels. Karami et al. [49] developed a synthetic procedure to synthesize chromophore-doped graphitic C-dots with d_av_ = 5 nm via a hydrothermal treatment of glucose and 3-nitroaniline dissolved in sulfuric acid. The C-dots exhibited three λ_ex_-independent emissions (blue, red and yellow) under acidic, neutral and alkaline environments, respectively. At pH = 2 the C-dots showed λ_max_ = 385 nm (QY = 43.0%), but at pH = 3 a weak red peak appeared along with the dominant blue peak. Upon increasing the pH, the intensity of the blue emission peak declined, whereas the red emission peak was enhanced, and at pH = 7 there was a single emission peak at λ_max_ = 610 nm (QY = 14.4%), which at pH =12 shifted to λ_max_ = 565 nm (QY = 24.3%). FT-IR spectra showed peaks related to O–H and N–H groups, C–H stretching vibrations, the C=O band of amide and carbonyl groups, C=C stretching/N–H bending bands and C–S/C–N/C–O stretching vibration bands. At the same time, a pH-dependent FT-IR peak centred at 1545 cm^−1^ was attributed to the presence of azo groups (–N=N), showing maximum intensity at neutral pH and minimum intensity at acidic pH.

#### 2.1.2. Extensive Conjugation Length

Early studies indicated the crucial role of quantum confinement in π-bonded hexagonal carbon clusters on the broadband emission of carbon-based films [50]. More recently, it has been shown that the HOMO–LUMO gap energy decreases as the size of GQDs increases [51]. Moreover, it has been demonstrated that the emission wavelength shows a linear size dependence in defect-free, functionality-free, zigzag-edged GQDs and that the fluorescence emission covers a broad spectrum from deep UV (λ_em_ = 235.2 nm) to NIR (λ_em_ = 999.5 nm) as the size varies from 0.46 nm to 2.31 nm [52]. At the same time, the presence of armchair edge widens the band gap in and blue shifts the emissive signals of GQDs [53].

**Figure 4:**
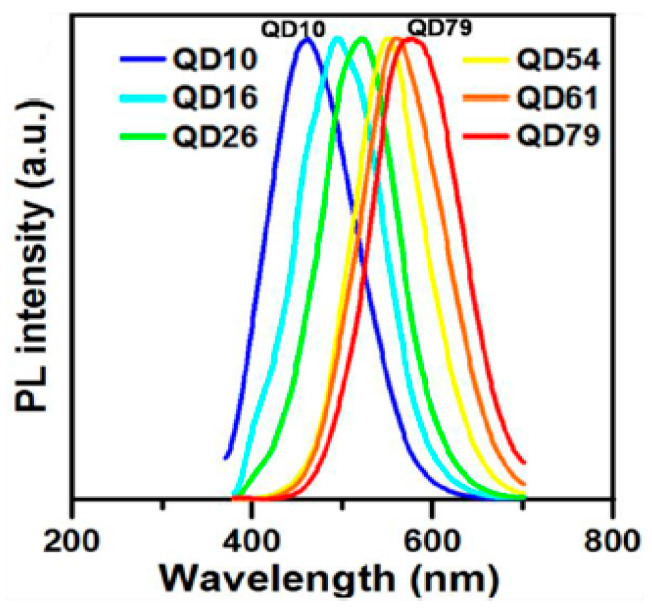
Normalized PL spectra (λ_ex_ = 350 nm) of QD10, QD16, QD26, QD54, QD61 and QD79. Reprinted (adapted) with permission from [54]. Copyright 2021 American Chemical Society.

Yeh et al. [54] relied on the oxidation of GO sheets to generate GQDs that were separated (via polyethersulfone membranes with different cutoff molecular weights) to fractions with d_av_ = 1.0 nm (QD10), 1.6 nm (QD16), 2.6 nm (QD26), 5.4 nm (QD54), 6.1 nm (QD61) and 7.9 nm (QD 79), all of which exhibited excitation-wavelength independent PL with λ_max_ at 460 nm, 490 nm, 520 nm, 540 nm, 560 nm and 580 nm, respectively (Figure 4).

**Figure 5:**
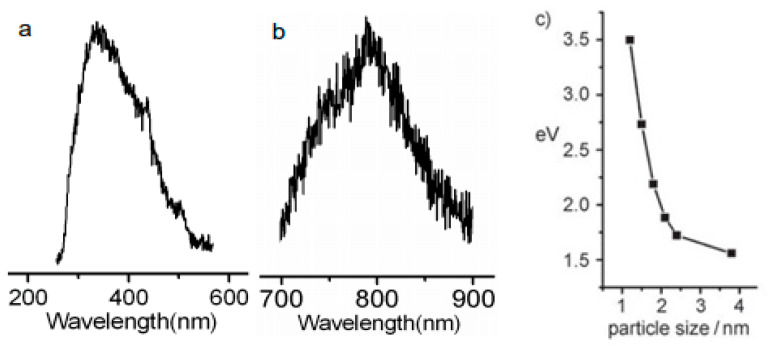
PL spectra of (**a**) small-sized C-dots (1.2 nm) and (**b**) large-sized C-dots (3.8 nm). (**c**) Size dependence of the bandgap in C-dots. Reprinted (adapted) with permission from [51]. Copyright 2021 John Wiley and Sons.

Li et al. [51] explored alkali-assisted electrooxidation of a graphite rod by applying different current densities to fabricate a series of C-dots and demonstrated that small- (1.2 nm), medium- (1.5–3 nm) and large-sized (3.8 nm) C-dots emit within the UV (350 nm), visible (400–700 nm) and NIR (800 nm) regions, respectively (Figure 5a,b). FT-IR spectroscopy indicated the presence of carbonyl (C=O) groups on the surface of these C-dots that can be eliminated via hydrogen plasma treatment without altering the PL properties of C-dots, an effect that points to the dominant role of quantum confinement for this class of materials. Figure 5c suggests that as the size of the C-dots increases, the HOMO–LUMO energy gap decreases, and therefore the PL emission is shifted towards the NIR region of the spectra.

Yuan et al. [55] followed solvothermal treatment of CA and diaminonaphthalene in ethanol and allowed different reaction times in the presence of concentrated sulfuric acid, yielding GQDs with d_av_ = 1.9 nm, 2.4 nm. 3.8 nm, 4.9 nm and 6.7 nm emitting respectively within the blue, green, yellow, orange and red regions, in agreement with the expected behaviour owing to quantum confinement effects. Similar trends were confirmed by Tian et al. [56], who explored the solvothermal treatment of CA and urea in water, glycerol, DMF and their mixtures to generate C-dots with tuneable size and PL emissions.

#### 2.1.3. Surface Functionalization and Passivation

Surface functionalization strategies have been shown to dramatically modify the PL properties of C-dots via defect and intrinsic state emission mechanisms [57] and charge transfer effects [58]. In principle, the presence of functional groups on C-dots generates structural defects and midgap states facilitating π*→midgap states→π transitions, thus enhancing the red components on their PL spectra [59]. An interesting study showed that within a series of C-dots with similar size distribution and graphitization degree, the bandgap was gradually reduced upon increasing the degree of surface oxidation, leading to a pronounced red shift from 440 to 625 nm [60]. In electrochemically oxidized C-dots it was shown that the PL mode stemming from the organic fluorophores was blue-shifted, while the PL component originated by the carbogenic cores was red-shifted [61].

**Figure 6:**
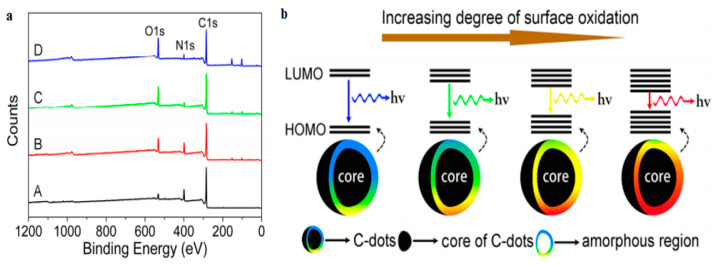
(**a**) XPS spectra of the fractions A, B, C and D. (**b**) Effect of the surface oxidation degree on the PL properties of C-dots (as the energy gap gets narrower, the photons’ frequency decreases). Reprinted (adapted) with permission from [60]. Copyright 2021 American Chemical Society.

Xiong’s group [60] synthesized a series of graphitic C-dots via hydrothermal treatment of urea and p-phenylenediamine (p-PD) yielding a mixture of four fractions (A, B, C and D in Figure 6a) that could be separated via silica column chromatography because of their different polarities. TEM imaging indicated that all fractions consisted of C-dots with d_av_ = 2.6 nm, while FT-IR spectra revealed the presence of O–H, N–H, C–O and –COOH groups on their surfaces. XPS indicated a systematic enhancement of the oxygen content when moving from fraction A to D, a trend that is accompanied by a systematic shift from blue to red PL emission at λ_ex_ = 365 nm. It has been suggested that as the population of surface oxygen atoms increases, the HOMO–LUMO bandgap gradually narrows, ultimately facilitating red PL emission (Figure 6b).

Liu et al. [62] followed a microwave-assisted pyrolysis of m-PD, o-PD and p-PD in formamide to generate three types of C-dots that exhibited λ_ex_-independent emission with λ_max_ at 360 nm, 450 nm and 530 nm, respectively. On the basis of FT-IR and XPS data, the authors demonstrated that the red-shifted C-dots exhibited a higher level of surface oxidation and possessed a larger population of amino groups on their surface. Zhang et al. [63] reported that blue-emitting C-dots (derived via pyrolysis of polyethylene glycol (PEG)) can be turned into green- and orange-emissive systems when treated thermally with PEG and 2,20-(ethylenedioxy)-bis(ethylamine), respectively.

Pang et al. [64] suggested that the PL of C-dots is directly related to surface-state emission, but the energy gap is controlled not only by the nature of the surface, but also by the size of the carbogenic core. In particular, they reported the synthesis of C-dots via HNO_3_-assisted oxidation of carbon fibres (CFs). By means of ultrafiltration and using membranes with molecular cutoffs < 3 kDa, 3–10 kDa and 10–30 kDa, three different fractions were isolated with d_av_= 2.7, 3.3 and 4.1 nm, respectively. Evidently, low concentrations of HNO_3_ facilitate the commencement of exfoliation from the defect sites of CFs, leading to small and blue-shifted C-dots. In contrast, higher concentrations of HNO_3_ and larger oxidation times give rise to larger, red-shifted C-dots with a higher degree of surface oxidation. By accurately controlling the oxidation conditions (reaction time, size of cutoff membranes, concentration of nitric acid), a series of well-defined C-dots were prepared that exhibited λ_ex_-independent emission, but their λ_max_ varied from 430 nm to 610 nm (λ_ex_ = 360 nm).

#### 2.1.4. Solvatochromism

Solvatochromism effects are common in C-dot dispersions and have been attributed to extensive nanoparticle–solvent dipole–dipole interactions and H-bonding [65]. By virtue of their highly responsive nature, solvatochromic C-dots have been explored as sensitive optical sensors to detect volatile organic compounds [66]. Interestingly, emissive contributions arising from edge and surface bands might induce solvatochromic shifts in the opposite directions [67]. At the same time, pyrrolic nitrogen- and amino nitrogen-enriched C-dots show wider solvatochromic shifts and impart higher QY [68].

Wang et al. [69] used p-PD and diphenyl ether as precursors to synthesize spherical C-dots with d_av_ = 2.6 nm. FT-IR revealed the presence of –OH, N–H, C–H, C=N, C=C, C–N and C–O groups. C 1s XPS indicated the presence of C–C/C=C, C–N, C–O and C=N; N 1s XPS indicated the presence of pyridinic, amino and pyrrolic N and O 1s XPS suggested the presence of C–O bonds. When the C-dots were dispersed in CCl_4_, toluene, CHCl_3_, acetone, DMF, CH_3_OH and H_2_O, their λ_max_ was at 511, 525, 545, 554, 568, 602 and 615 nm, respectively. In other words, the PL of C-dots systematically red-shifts as the polarity of the aprotic solvent increases. In protic solvents (such as methanol, ethanol, benzyl alcohol, 1-hexanol and 1-octanol), the C-dots emitted identical red PL regardless of the nature of the solvent, an effect that has been attributed to the formation of hydrogen bonds between the N and O atoms on the surface of C-dots and the -OH groups of the solvent molecules, which stabilize the excited states.

**Figure 7:**
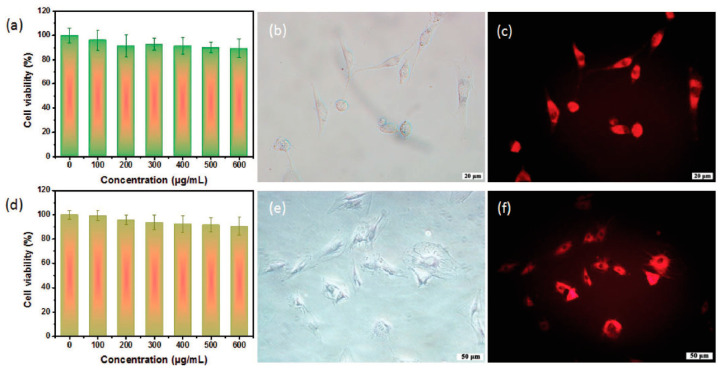
Viability of MC3T3-E1 (**a**) and BMSCs cells (**d**) after incubation with C-dots for a 24 h uptake process. FL images of (**b**,**c**) MC3T3-E1 and (**e**,**f**) BMSCs, respectively; (**b**,**e**) bright field and (**e**,**f**) under λ_ex_ = 530 nm. Reprinted (adapted) with permission from [72]. Copyright 2021 John Wiley and Sons.

In addition, Li et al. [70] followed thermal treatment of CA, DMF and urea to synthesize C-dots that showed enhanced red (λ_max_ =640 nm) and NIR (λ_max_ =760 nm) emission in aprotic solvents such as DMSO, DMF and N-methyl-2-pyrrolidone. However, in water, these C-dots exhibited weaker signals in the red region and showed no NIR emission. This solvatochromic effect was ascribed to electron-acceptor groups (S=O/C=O) of the aprotic solvents attached to the outer layers and the edges of the C-dots. Yang et al. [71] synthesized triphenylphosphine (P(Ph)_3_) functionalized C-dots (p-C-dots) with d_av_ = 4 nm and QY up to 58%. It was observed that λ_ex_ of p-C-dots systematically red-shifted from 431 to 641 nm upon increasing the dielectric constant (ε) of the dispersion medium (Figure 7), an effect that has been attributed to the reduced electron-donating capacity of the P(Ph)_3_ groups in environments with increased polarity.

### 2.2. Applications of Aqueous Dispersions of Red C-Dots

#### 2.2.1. Bioimaging

Red/NIR-emissive C-dots are considered to be ideal for bioimaging applications because they allow larger penetration depth without damaging the surrounding tissue. To that end, Liu et al. [72] suggested a rapid synthesis of red-emitting C-dots (λ_max_ = 630 nm) with QY = 10.8% in water and 31.5% in ethanol (λ_ex_ = 540 nm) using o-PD as a precursor in diluted HNO_3_ via a one-step hydrothermal procedure. The C-dots showed low level of toxicity against mice osteoblasts MC3T3-E1 and bone marrow stromal cells (BMSCs) (Figure 7a,d). The bright field and FL images (λ_ex_ = 530 nm) of MC3T3-E1 and BMSCs incubated with C-dots at a concentration of 200 μg/mL for 24 h at 37 °C are displayed in Figure 7b,c,e,f, suggesting that C-dots are predominantly distributed into the cytoplasm and not the nucleus, thus facilitating high resolution cell imaging without affecting the replication or the transcription of DNA.

Tan et al. [73] followed electrochemical exfoliation of graphite in 0.01 M K_2_S_2_O_8_ solution to synthesize red C-dots with d_av_ = 3 nm and λ_max_ = 610 nm at λ_ex_ = 500 nm. Those C-dots, when used without any further surface treatment, showed minimal toxicity against HeLa cells and were able to stain both their cell membranes and their cytoplasm, offering clear images under an FL microscope while exhibiting excellent photostability over a prolonged period.

#### 2.2.2. Sensing/Biosensing

**Figure 8:**
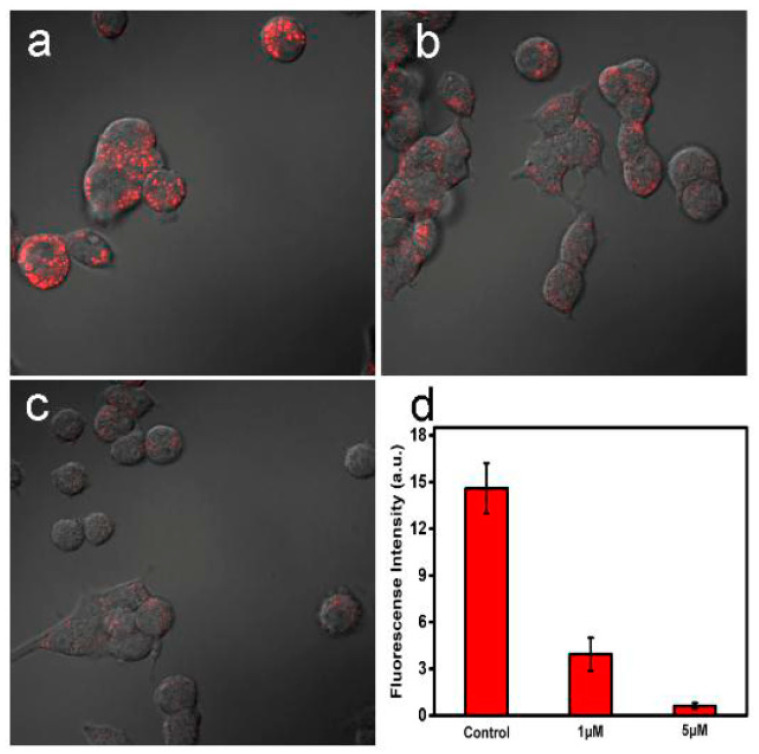
FL images of living PC12 cells stained with 5.0 µM S,N-C-dots: (**a**) in the absence of Fe^3+^ (control sample), (**b**) in the presence of 1.0 µM Fe^3+^, (**c**) in the presence of 5.0 µM Fe^3+^. (**d**) The PL intensity of samples described in (**a**–**c**). Reprinted (adapted) with permission from [74]. Copyright 2021 Elsevier.

Oftentimes, heteroatom doping is coupled with surface patterning strategies and sp^2^ domain engineering in order to optimize red-shifted emissions. Along those lines, Li et al. [74] followed hydrothermal treatment of CA and thiourea to synthesize red-emitting S,N codoped C-dots that were functionalized with phenylboronic acid tags yielding S,N-C-dots-PBA with QY = 23% and λ_max_ = 593 nm under λ_ex_ = 550 nm. The S,N-C-dots-PBA showed excellent biocompatibility and were able to illuminate PC12 cells under the FL microscope 20 min after their injection (Figure 8a). Interestingly, the PL intensity decreased upon the addition of 1.0 and 5.0 µM Fe^3+^ (Figure 8b,c), and the corresponding intracellular PL was compared to the S,N-C-dot-free sample (control) (Figure 8d). The quenching efficiency was 73% and 96% for 1.0 µM and 5.0 µM Fe^3+^ ions, respectively. This behaviour indicates that S,N-C-dots-PBA can be explored for the highly sensitive intracellular detection of Fe^3+^.

**Figure 9:**
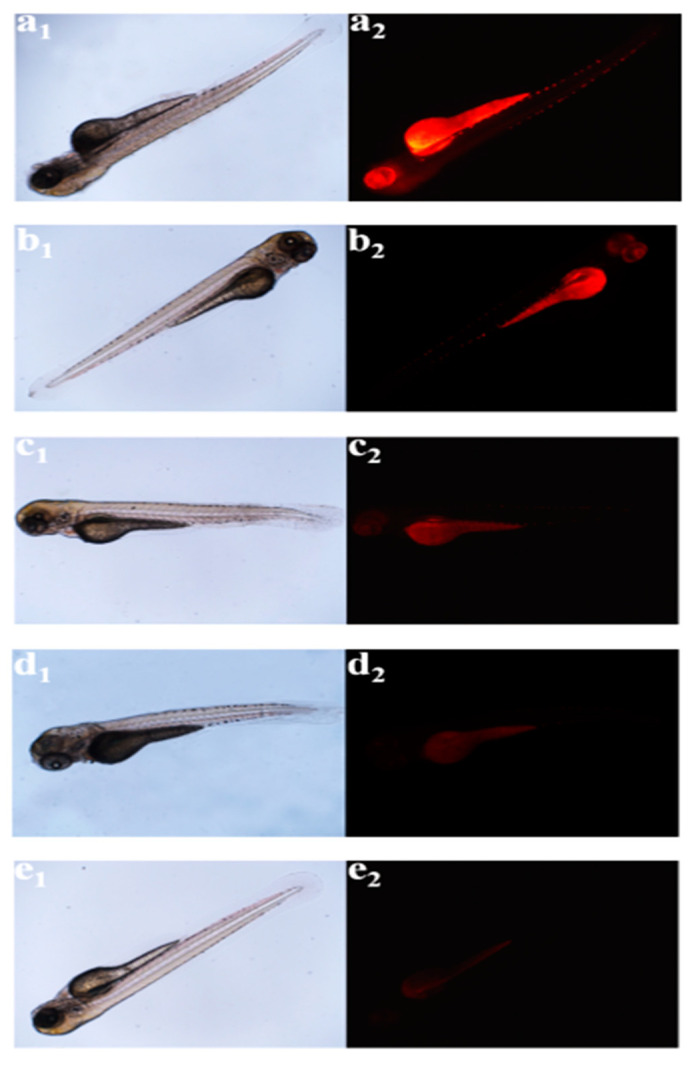
FL imaging of Pt^2+^ in ZF: (**a_1_**–**e_1_**) Optical images and (**a_2_**–**e_2_**) FL images stemming from C-dots in ZF loaded with several concentrations of Pt^2+^: 0 (**a_1_**,**a_2_**); 30 (**b_1_**,**b_2_**); 60 (**c_1_**,**c_2_**); 100 (**d_1_**,**d_2_**) and 150 μM (**e_1_**,**e_2_**). Reprinted (adapted) with permission from [75]. Copyright 2021 American Chemical Society.

In addition, Gao et al. [75] followed hydrothermal treatment of CA and neutral red dye (NR) (ratio of 1000:1) to prepare red-emissive C-dots, the PL of which quenched in the presence of Pt^2+^ (ethanol as solvent) due to a rapid electron-transfer process between the metal ions and the C-dots’ surface. Moreover, zebrafish (ZF) that were loaded with Pt^2+^ and C-dots exhibited PL properties in a manner that critically depended on the concentration of the internalized Pt^2+^ (Figure 9), indicating that C-dots can be used as sensors to quantify the Pt^2+^ levels present in living organisms.

#### 2.2.3. Photothermal Therapy (PTT)

Photothermal therapy (PTT) relies on radiation-absorbing particles to cause thermal ablation of tumour cells and subsequent cell death. Red-shifted C-dots are ideal candidates as PTT agents due to their low phototoxicity, great photothermal conversion effectiveness and deep tissue penetration.

**Figure 10:**
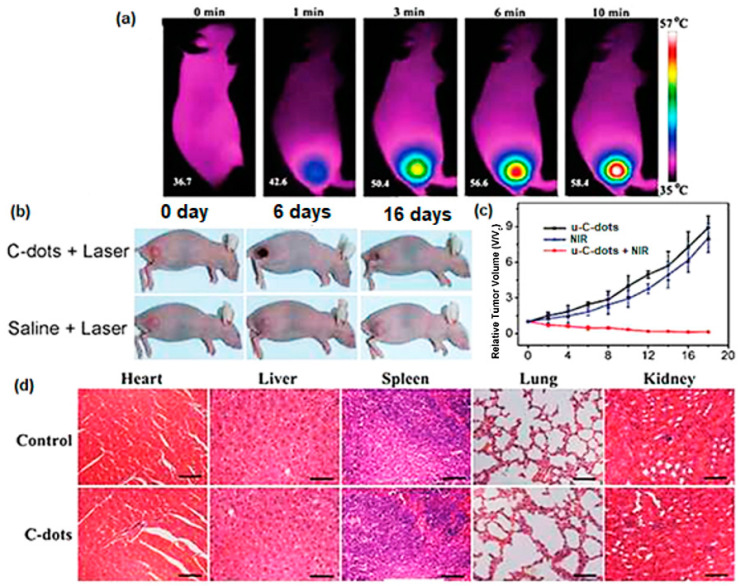
(**a**) IR images of u-C-dots injected into mice tumour locations under laser irradiation (671 nm at 2 W/cm^2^); (**b**) photos of the mice tumours on several days after the two treatments; (**c**) relative change in the tumour volume over time during the treatments utilizing only u-C-dots, only NIR irradiation and their combination; (**d**) haematoxylin- and eosin-stained slices of the heart, liver, spleen, lung and kidney in mice after PTT. Scale bar is 50 μm. Reprinted (adapted) with permission from [47]. Copyright 2021 John Wiley and Sons.

As mentioned in the *Heteroatom doping* section, Ge et al. [47] developed a hydrothermal approach of PPA to fabricate red-emissive C-dots (u-C-dots) that showed a wide absorption range from 400 to 750 nm and 38.5% photothermal conversion. Those properties make u-C-dots suitable for PL and photoacoustic (PA) imaging as well as PPT. In Figure 10a IR images of a mouse that was subjected to intertumoral injection of u-C-dots indicate that the temperature of the tumour cells rose up to 58.4 °C within 10 min of laser radiation, thus causing permanent damage to cancer cells. Following a 16-day course, the tumour disappeared and the mouse fully recovered, while the mouse receiving saline treatment failed to do so (Figure 10b,c). At the same time, no distinct inflammation, cell necrosis or apoptosis in the heart, liver, spleen, lung or kidney were observed, confirming the absence of undesired side effects induced by u-C-dots (Figure 10d).

#### 2.2.4. Photodynamic Therapy (PDT)

Photodynamic therapy (PDT) has distinct advantages over traditional cancer treatments (surgery, chemotherapy and radiotherapy) such as low toxicity, minimal damage to healthy tissue and mild side effects. In PDT, a laser beam excites the photosensitizer to generate reactive oxygen that eventually destroys the tumour cells.

**Figure 11:**
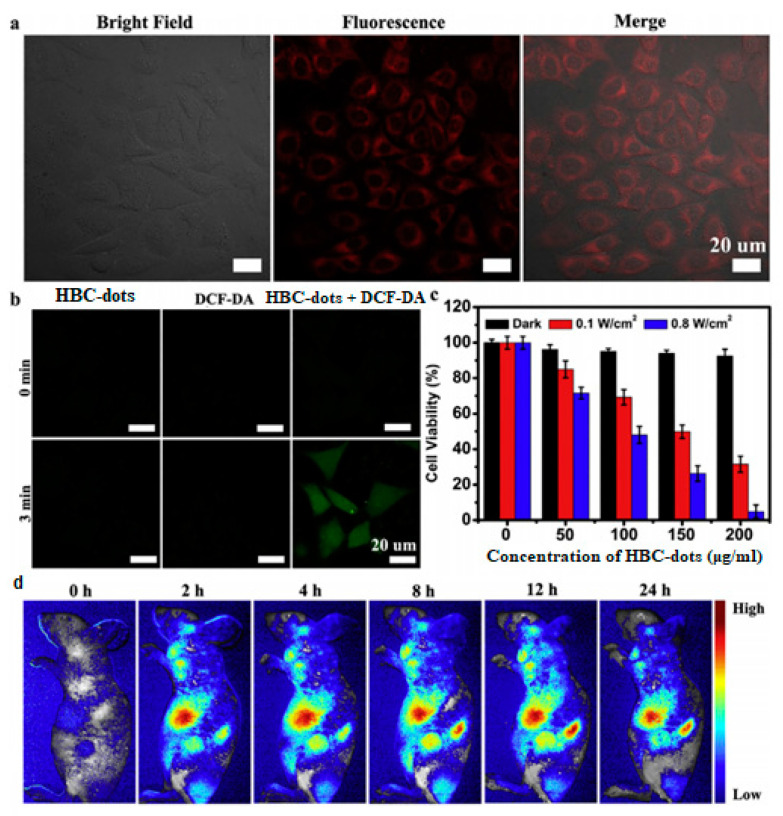
(**a**) FL images of HeLa cells incubated with HBC-dots. (**b**) FL imaging of HeLa cells stained with HBC-dots, DCF-DA and their combination. (**c**) Viability of HeLa cells incubated with HBC-dots after several treatments. (**d**) In vivo FL images of mice after intravenous injection of HBC-dots in PBS. Reprinted (adapted) with permission from [76]. Copyright 2021 Elsevier.

Jia’s group [76] followed solvothermal treatment of *Hypocrella Bambusae* to synthesize red-emissive HBC-dots with d_av_ = 4.8 nm and λ_max_ = 610 nm at λ_ex_ = 540 nm. After 10 min of laser irradiation (635 nm at 0.8 W/cm^2^), the temperature of the HBC-dot solution (200 μg/mL) rose by 26.9 °C, while it increased by only 3.7 °C upon irradiation at 0.1 W/cm^2^. The results confirmed that the HBC-dots, at 0.1 W/cm^2^, could produce ^1^O_2_, facilitating PDT, but at 8 W/cm^2^ they could generate both ^1^O_2_ and thermal energy, facilitating both PDT and PTT treatments. Upon a 6-hour incubation of HeLa cells with HBC-dots, it was observed that the cytoplasm emitted red PL (Figure 11a). The coincubation of HeLa cells with HBC-dots and 2′,7′-Dichlorofluorescin diacetate (DCF-DA), a green dye that is commonly used to probe intracellular ^1^O_2_ formation, followed by 635 nm irradiation gave rise to strong green PL emission (Figure 11b), thus confirming the presence of ^1^O_2_. Moreover, standard MTT assay showed that HBC-dots exhibited low toxicity against HeLa cell under dark conditions (black bars in Figure 11c). The cell viability was compromised at power density at 0.1 W/cm^2^ (PDT system, red bars in Figure 11c), and the effect was more pronounced at power density at 0.8 W/cm^2^ (PDT+PTT system, blue bars in Figure 11c), given that 99% of HeLa cells incubated with 200 mg/mL HBC-dots did not survive. After intravenous injection of HBC-dots in mice, the PL intensity in cancer sites rose with time, reaching its maximum value after 8 h (Figure 11d).

Ge et al. [77] synthesized C-dots with excellent water solubility using polythiophene derivatives as the precursor via a hydrothermal method. The C-dots showed interesting properties such as a broad absorption region from the visible to the NIR, superior biocompatibility and photostability, high ^1^O_2_ yield and strong emission with λ_max_ = 680 nm at λ_ex_ = 488 nm.

### 2.3. Solid-State Red C-Dots and Their Applications

Significant progress has been made in solid-state red C-dot-based systems such as hybrid powders and polymer nanocomposites typically derived via in situ polymerization, mechanical and melt mixing and solution blending.

#### 2.3.1. WLEDs (White Light-Emitting Diodes)

**Figure 12:**
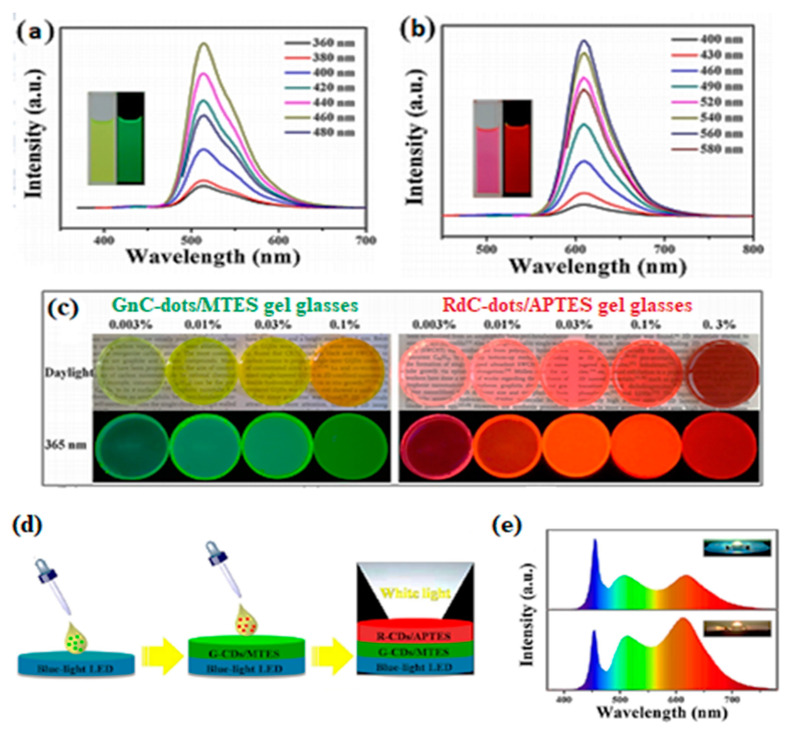
PL spectra of (**a**) GnC-dots and (**b**) RdC-dots under different λ_ex_ (inset: images under daylight (left) and 365 nm light (right)). (**c**) Photographs of GnC-dot/MTES and RdC-dot/APTES gels bearing various C-dot loadings under daylight (up) and 365 nm excitation (down). (**d**) Fabrication of WLEDs from GnC-dot/MTES and RdC-dot/APTES gel glasses. (**e**) EL spectra (up: cold WLED, down: warm LED and insets: the picture below WLED). Reprinted (adapted) with permission from [78]. Copyright 2021 American Chemical Society.

Yuan et al. [78] fabricated graphitic, excitation-independent, green-emitting GnC-dots (d_av_ = 6.5 nm, λ_max_ = 515 nm, λ_ex_ = 460 nm, QY = 81%) following solvothermal treatment of perylene that were further treated with NaOH to produce red-emitting RdC-dots (d_av_ = 6.45 nm, λ_max_ = 610 nm, at λ_ex_ = 560 nm, QY = 80%), as shown in Figure 12a,b. FT-IR proved the presence of –NO_2_ and –NH_2_ for the GnC-dots and the C=O in the quinone structure for the RdC-dots. The dispersion of GnC-dots into methyltriethoxysilane (MTES) and RdC-dots into 3-triethoxysilylpropylamine (APTES) gave rise to transparent nanocomposite gel glasses that under UV radiation appeared green and red, respectively (Figure 12c). To form WLEDs, the GnC-dots/MTES gel was deposited (and allowed to dry) on a blue LED chip (27 lm/W at 20 mA, λ_ex_ = 460 nm), followed by the deposition of RdC-dots/APTES gel (Figure 12d). The electroluminescence (EL) spectra of both cold and warm WLED lamps revealed three emission peaks located at 460, 508 and 615 nm, corresponding to the emissions of the LED chip, GnC-dots and RdC-dots (Figure 12e), respectively. Moreover, the warm WLED lamp showed a high colour rendering index (CRI) of 92.9, compared to CRI = 81.1 for the cold lamp.

Jin et al. [79] followed hydrothermal treatment of a l-tyrosine, o-PD, l-tyrosine/o-PD mixture to generate blue (d_av_ =5.1 nm, QY = 8.6% at λ_ex_ = 365 nm), green (d_av_ = 5.7 nm, QY = 12.6% at λ_ex_ = 430 nm) and orange/red C-dots (d_av_ = 4.4 nm, 20.9% at λ_ex_ = 405 nm). Subsequently, the C-dots were dispersed in polyvinyl alcohol (PVA) to generate blue-, green- and orange/red- emissive films. The films were deposited on a UV chip to generate WLED with *Commission Internationale de L’Eclairage* (CIE) chromaticity coordinates (0.30, 0.33), compared to pure white light (0.33, 0.33). 

As aforementioned, Wang et al. [69] followed a solvothermal treatment of p-PD into diphenyl ether to synthesize graphitic C-dots that exhibited λ_max_ at 511, 525, 545, 554, 568, 602 and 615 nm in CCl_4_, toluene, CHCl_3_, acetone, DMF, CH_3_OH and H_2_O, respectively. To prepare liquid LEDs, C-dot dispersions in toluene, DMF and methanol were encapsulated in silica glass boxes and packed above a UV-LED chip (λ_em_ = 370 nm) in order to generate green, yellow and red PL contributions respectively. Moreover, the green-emissive C-dots in methyl methacrylate nanocomposite and the red-emissive C-dots in PVA nanocomposite were deposited on a UV-LED chip to assemble solid LEDs.

Zhang et al. [80] synthesized a colour-tuneable solid-state luminescent material via a hydrothermal procedure using CA, urea and Eu (DPA)_3_ and a 2D-layered-structure nanoclay. The hybrid material obtained showed tuneable emission colours from red to blue under different λ_ex_, where the Eu^3+^ and C-dots mainly contributed to the red and blue emission, respectively, making them potential candidates for WLED applications. Yuan et al. [81] synthesized amorphous, red-emissive C-dots using 1,2,4-triaminobenzen as carbon source and PEG200 as passivation agent via a solvothermal method. The C-dots showed λ_ex_-independent behaviour, with a QY up to 25% in ethyl acetate (λ_ex_ = 460 nm), and were combined with silica to fabricate red PL powder in order to construct warm WLEDs.

#### 2.3.2. Pollutant Sensing

**Figure 13:**
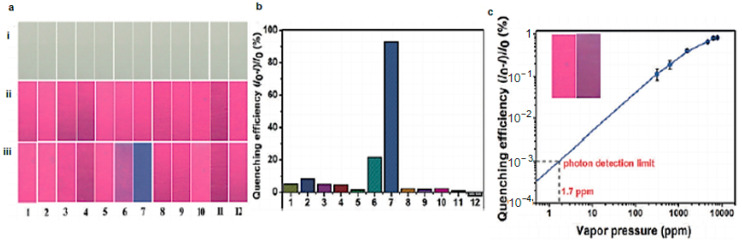
(**a**) Images of PVDF/C-dot membranes under visible light (i) and before (ii) and after (iii) exposure to saturated vapours of various solvents with a response time of 30 s (λ_ex_ = 365 nm): 1, acetonitrile; 2, o-nitrotoluene; 3, toluene; 4, cyclohexylamine; 5, hydrazine; 6, ethanediamine; 7, ammonia; 8, dimethyl sulfoxide; 9, acetic acid; 10, acetone; 11, chloroform; 12, hydrochloric acid. (**b**) Quenching efficiency in the presence of molecules 1–12. (**c**) Quenching efficiency as a function of vapour pressure of ammonia. Inset: Images of the PVDF/C-dots before (**left**) and after (**right**) exposure to 310 ppm of ammonia with a response time 30 sec (λ_ex_ = 365 nm). Reprinted (adapted) with permission from [82]. Copyright 2021 John Wiley and Sons.

Liang’s group [82] followed the carbonization of sugarcane bagasse in a mixture of concentrated sulfuric and phosphoric acid to synthesize red-emitting C-dots that were subsequently coated on polyvinylidene fluoride (PVDF) membranes. The PVDF/C-dot membranes exhibited red PL (λ_ex_ = 365 nm) that was quenched in the presence of ammonia, but not in the presence of acetonitrile, o-nitrotoluene, toluene, cyclohexylamine, hydrazine, ethanediamine, dimethyl sulfoxide, acetic acid, acetone, chloroform or hydrochloric acid (Figure 13a,b). The PVDF/C-dot system was shown to be a highly selective and highly sensitive sensor for ammonia with a detection limit of 1.7 ppm and a response time of 30 s (Figure 13c).

Lu et al. [83] followed hydrothermal treatment of citric acid and ethylenediamine in formamide−water binary systems to generate a mixture of blue and red-emissive C-dots. Subsequently, ion imprinted mesoporous polymers were prepared using Cr^3+^ and Pb^2+^ as templates and the mixture of blue and red C-dots as fluorescent probes to generate a dual channel detection system, given that Cr^3+^ only quenches the emission of blue C-dots and Pb^2+^ only quenches red C-dots. The detection limits for Cr^3+^ and Pb^2+^ were 27 nm and 34 nm, respectively. Hu et al. [84] followed a solvothermal treatment of o-phenylenediamine and selenourea in HCl to synthesize red-emissive Se,N,Cl codoped C-dots with QY = 23.6%, the emission of which can be selectively quenched in the presence of malachite green (MG), thus functioning as nanosensors for MG with a limit of detection close to 20 nM.

#### 2.3.3. Nanoforensics

**Figure 14:**
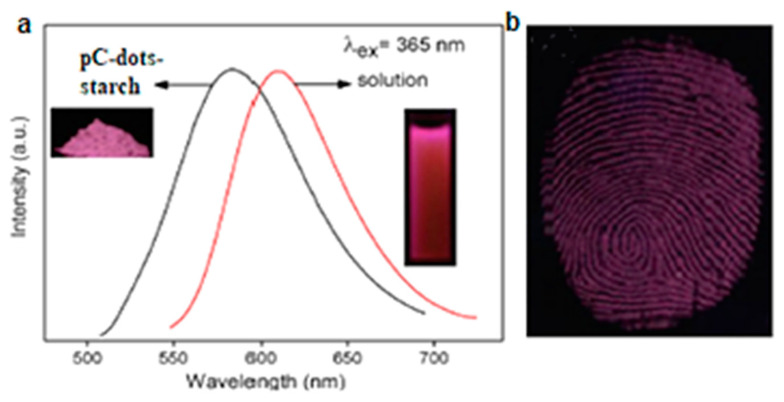
(**a**) PL emission spectra of pC-dots in ethanol and pC-dot starch powder. Inset: pC-dots in ethanol solution (**right**) and pC-dot starch powder (**left**) (λ_ex_ = 365 nm). (**b**) Image of fingerprints developed with pC-dot starch on glass substrate (λ_ex_ = 365 nm). Reprinted (adapted) with permission from [83]. Copyright 2021 Elsevier.

Li et al. [85] synthesized red-emissive, λ_ex_-independent, graphitic C-dots (pC-dots) with d_av_ = 12.5 nm following solvothermal treatment of p-PD. FT-IR of pC-dots showed the stretching vibrations of N–H, C=N, C=C and C=C, and XPS spectra revealed they consisted of C (79.56%), N (15.61%) and O (4.83%). As shown in Figure 14a, incorporation of pC-dots into starch gave rise to highly PL powders (the PL spectra of pC-dots in ethanol is also displayed for comparison). The pC-dot/starch powder was used to develop latent fingerprints deposited on a glass substrate and was able to reveal well-defined ridges with minimal background interference (Figure 14b).

Yuan et al. [86] followed a solvothermal treatment of o-phenylenediamine and 1,8-diamino naphthalene in DMF in the presence of boric acid to synthesize red-emissive B-doped C-dots with QY = 12%. The B-C-dot dispersion in PVA was used as an ink for advanced anticounterfeit applications, given that it appears blue upon UV excitation but becomes pink after alkali treatment and returns to blue after acid treatment in a reversible manner.

## 3. Conclusions

Although the large-scale production of well-defined, red-emissive C-dots remains an open challenge, a number of promising approaches towards this direction have been demonstrated. These synthetic strategies rely on heteroatom doping, surface functionalization and the formation of extensively conjugated domains, while a combination of two or more of those engineering principles is usually more effective in terms of PL performance. In contrast to their blue and green counterparts, the synthesis of red C-dot-based systems is rather tedious and energy intensive and largely depends on the use of aromatic precursors, organic solvents and toxic compounds. Overcoming those barriers will allow the development of a new generation of photoactive materials that can potentially advance crucial biomedical treatments, sensing applications and lighting technologies.

## 4. Outlook

There is no doubt that C-dots are a highly promising class of nanomaterials with distinct advantages with respect to conventional organic fluorescent dyes because of their multicolour nature and enhanced structural stability, while they are superior in terms of environmental friendliness and preparation ease compared to heavy metal-based quantum dots. These nanoemitters can be used in isolation for a variety of applications [38] or can be combined with other materials to generate advanced nanocomposites and devices, such as polymeric hybrids [27], powder compositions [87], ordered mesoporous frameworks [88] and C-dot-based Forster resonance energy transfer systems [89]. At the same time, their photoluminescence mechanism is not fully understood, and more studies are needed in order to gain further insights on the structure–properties relationships of those complex systems. This observation is particularly true for red-emissive C-dots, which are only recently gaining significant attention from the scientific community, although both challenges and benefits associated with their production are well-recognized. 

First is the observation that the majority of C-dots typically display weak emissions on the red end of the spectrum, while certain approaches allow the synthesis of predominantly red nanoemitters. A recent theoretical study indicated that improved red emission is expected for C-dots bearing abundant weakly interacting surface emission centres [90]. Simple post-modification treatments (such as the reaction with acetaldehyde [91]) can modulate the PL emission, essentially converting blue-emitting into red-emitting C-dots. In addition, C-dots with reversibly switchable green–red emissions have been reported [78,86].

Second is the realization that a number of effective strategies for the large-scale production of blue and green C-dots that require a relatively low capital investment are emerging in the literature, but those efforts are less advanced for their red-emissive counterparts. Nevertheless, cost-effective approaches that are compatible with standard industrial processing are present in the literature. For example, far-red C-dots with QY = 18.5% were prepared via a 3 min microwave treatment of glutathione in formamide followed by centrifugation and dialysis [92]. Moreover, red C-dots with QY = 53% can be produced on a gram scale by heating a formamide solution of citric acid and ethylenediamine [93].

Third is the understanding that the nature of the precursor material plays a significant role on the PL properties of the red-emissive C-dots. For example, red C-dots with QY = 84% are derived from the highly conjugated molecular precursor tris(4-aminophenyl)amine [94]. In analogy to blue and green C-dots, organic fluorophores developed in situ during the synthesis of red C-dots are expected to have a pronounced impact on their PL properties. We note that it has been suggested that red emission might be associated exclusively to organic fluorophores entrapped within the carbogenic structure [37]. In terms of biomass utilization, we note that red-emitting N,Mg codoped C-dots from the leaves extract of Bougainvillea plant showed λ_ex_-independent emissions with λ_max_ = 678 nm and QY = 40% [95], while NIR-C-dots with QY = 31% and 15.3% were synthesized by heating a formamide solution of lemon juice [40] and via solvothermal treatment of spinach [96].
